# Polysaccharides from Citrus Fruit with Different Mastication Traits Ameliorate DSS-Induced Ulcerative Colitis by Restoring Intestinal Barrier Function and Microbiota Balance

**DOI:** 10.3390/foods15010052

**Published:** 2025-12-24

**Authors:** Jieqiong Yao, Siyi Pan

**Affiliations:** 1 College of Food Science and Technology, Huazhong Agricultural University, Wuhan 430070, China; yaojieqiong2021@webmail.hzau.edu.cn; 2 Key Laboratory of Environment Correlative Dietology, Ministry of Education, Huazhong Agricultural University, Wuhan 430070, China; 3 Hubei Key Laboratory of Fruit & Vegetable Processing & Quality Control, Huazhong Agricultural University, Wuhan 430070, China

**Keywords:** mastication trait, citrus polysaccharides, ulcerative colitis, intestinal barrier, gut microbiota, short-chain fatty acids

## Abstract

Mastication trait is an important indicator for evaluating citrus fruit texture and may modulate the biological activity of citrus polysaccharides. This study compared the protective effects of pulp polysaccharides from citrus fruit with different mastication traits, namely Hongmeiren (HMR, superior), Satsuma (WM, moderate), and Nanfeng tangerine (NF, inferior mastication trait) on ulcerative colitis (UC) in mice. All polysaccharides alleviated UC symptoms, with HMR showing the most pronounced effects by more markedly reducing TNF-α levels, while enhancing IL-10, ZO-1, Occludin, and MUC2 expression. NF exhibited a stronger inhibitory effect on IL-6. Microbiota analysis revealed that citrus polysaccharides suppressed Escherichia–Shigella and Helicobacter, enriched *Akkermansia* and *norank_f_Muribaculaceae*, and promoted short-chain fatty acid production, particularly of propionate and butyrate. LEfSe analysis further indicated that HMR selectively enriched *Dubosiella* and *norank_o_Clostridia_UCG-014*, WM increased the abundance of *Lachnospiraceae_NK4A136_group*, and NF promoted the growth of *Parabacteroides*. Overall, HMR with superior mastication trait exhibited the greatest efficacy in attenuating inflammation and maintaining intestinal homeostasis. These findings reveal an intrinsic link between citrus mastication traits and their colitis-alleviating activity, offering a mechanistic basis for dietary intervention in inflammatory bowel disease as well as for functional citrus breeding.

## 1. Introduction

Ulcerative colitis (UC) is a chronic relapsing form of inflammatory bowel disease, characterized by persistent inflammation and mucosal ulceration of the colonic mucosa. Its principal clinical manifestations include diarrhea, abdominal pain, rectal bleeding, and weight loss [1]. Although its precise pathogenesis remains incompletely elucidated, it is generally recognized to be closely associated with host genetic susceptibility, compromised mucosal barrier function, gut microbiota dysbiosis, and environmental factors [2]. Currently, the clinical treatment of UC mainly relies on drugs such as corticosteroids, aminosalicylates, and immunosuppressants; however, long-term use often leads to drug resistance and multiple side effects [1]. Consequently, developing safe and sustainable natural intervention strategies has become an important direction for the prevention and adjunctive treatment of colitis. Dietary polysaccharides have received increasing attention due to their immunomodulatory, anti-inflammatory, and antioxidant bioactivities, as well as their potential to regulate the gut microbiota.

In recent years, accumulating studies have demonstrated that dietary polysaccharides exert significant protective effects against UC through multiple mechanisms, including suppression of inflammatory responses, restoration of the intestinal epithelial barrier, modulation of host immunity, and remodeling of the gut microbiota [3,4]. For instance, Han et al. [5] reported that polysaccharides from *Gracilaria lemaneiformis* significantly suppressed the expression of IL-1β and TNF-α in DSS-induced colitis mice while promoting the recovery of MUC2, ZO-1, and Occludin. Lv et al. [6] demonstrated that *Rehmannia glutinosa* polysaccharides rich in arabinose side chains effectively ameliorated tissue damage and colonic inflammation by reshaping microbial composition and restoring short-chain fatty acid (SCFA) levels. Furthermore, Jiang et al. [7] found that differences in the side-chain composition of kiwifruit pectic polysaccharides of varying ripeness levels resulted in distinct efficacies in alleviating colitis. In particular, pectin from mature kiwifruit more effectively restored ZO-1 expression, reduced IL-1β, IL-6, and TNF-α levels, and markedly enhanced butyrate production. Existing studies have primarily focused on plant polysaccharides with specific structural features, whose anti-inflammatory and gut-modulating effects have been widely documented. However, systematic studies on the regulatory effects of polysaccharides from fruits and vegetables with varying textural properties on UC remain lacking.

Citrus, a member of the Rutaceae family, is among the most important fruit crops globally. The mastication trait serves as a critical parameter for assessing citrus texture, reflecting the degree of pulp fragmentation during chewing and its swallowing characteristics, which directly influence consumer sensory preference and industrial processing performance [8]. The “inferior mastication trait” of citrus fruit is defined as having a coarse texture, with substantial fibrous residues remaining before swallowing even after thorough chewing [9]. Driven by market demand, improving mastication traits has become a major direction in citrus quality enhancement and breeding programs [10]. Our previous investigation of three representative citrus cultivars with different mastication traits, including Hongmeiren (HMR, superior mastication trait), Satsuma (WM, moderate), and Nanfeng tangerine (NF, inferior), revealed that NF exhibited higher cellulose content with markedly higher crystallinity, a denser intra- and inter-chain hydrogen-bonding network, and short, highly aggregated cellulose microfibrils, whereas HMR showed lower cellulose crystallinity and a looser fibrillar arrangement. In addition, NF possessed more extensively branched pectin with abundant arabinose- and galactose-rich RG-I side chains. In contrast, HMR contained a higher proportion pectin and less branched pectin. WM displayed intermediate characteristics between NF and HMR. These structural distinctions in both pectin architecture and cellulose organization underlie the divergent mechanical properties associated with their mastication traits [11]. In vitro colonic fermentation further indicated that citrus with different mastication traits could selectively modulate the composition of the gut microbiota. Among them, HMR, which possesses a superior mastication trait, exhibited a higher degree of carbohydrate degradation and greater SCFAs production, especially butyrate, which is an important metabolic substrate for maintaining colonic epithelial health. However, the physiological effects and underlying mechanisms by which mastication trait-driven variations in citrus polysaccharide composition and structure influence the prevention and mitigation of colitis remain unclear.

In this study, a DSS-induced UC mouse model was used to systematically compare the differences in the colitis-ameliorating effects of polysaccharides (HMR, WM, and NF) extracted from citrus pulp with distinct mastication traits. We hypothesized that structural differences in citrus polysaccharides driven by mastication traits result in distinct anti-colitic effects. Through evaluation of histopathological alterations, barrier function, inflammatory cytokines, oxidative stress levels, SCFAs content, and gut microbiota composition, the study elucidates their potential anti-inflammatory and microbiome-modulating mechanisms, thereby providing a scientific basis for the functional utilization and targeted breeding of citrus.

## 2. Materials and Methods

### 2.1. Preparation of Polysaccharides

Three citrus cultivars with different mastication traits, Hongmeiren (HMR, superior mastication trait), Satsuma (WM, moderate), and Nanfeng tangerine (NF, inferior), were harvested from experimental orchards in Ningbo, Danjiangkou, and Fuzhou, China, respectively. After peeling, the citrus pulp was homogenized, freeze-dried, and ground into fine powder. Polysaccharides were extracted according to the method described in our previous study [11]. In brief, 10 g of freeze-dried pulp powder was suspended in 500 mL of 95% ethanol, boiled for 20 min, and filtered. The extraction was repeated until no soluble sugars were detected by the phenol–sulfuric acid method. The resulting residue was incubated overnight at 4 °C in 90% dimethyl sulfoxide (DMSO) to remove starch, washed thoroughly with distilled water, and then treated with a methanol–chloroform mixture (1:1, *v*/*v*) to remove residual proteins. The sample was further washed twice with acetone and dried at 40 °C to obtain cell wall polysaccharides. The monosaccharide composition and structural characteristics of polysaccharides extracted from citrus with different mastication traits are presented in the Supplementary Materials.

### 2.2. Animal Experimental Design

Forty-eight male C57BL/6 mice (6 weeks old, 20 ± 2.0 g) were purchased from the Experimental Animal Center of Huazhong Agricultural University (Wuhan, China). The mice were housed under specific pathogen-free conditions in the center, maintained at 22 ± 2 °C with 40–70% relative humidity and a 12 h light/dark cycle. They were provided with standard laboratory chow and water ad libitum. All animal experimental procedures were reviewed and approved by the Scientific Ethics Committee of Huazhong Agricultural University (Approval No. HZAUMO-2025-0451, Approval Date: 13 November 2025).

After a one-week acclimatization period, the mice were randomly divided into six groups (n = 8): normal control (NC), model (DSS), positive control (5-ASA, 150 mg/kg), HMR (400 mg/kg), WM (400 mg/kg), and NF (400 mg/kg). Mice in the sample groups were administered the corresponding citrus polysaccharide solution by oral gavage daily at a dose of 0.1 mL/10 g body weight. The NC and DSS groups received an equivalent volume of sterile water. The treatment lasted for 21 days. Mice in the 5-ASA group received sterile water during the first 14 days, followed by 5-ASA solution at the same volume for days 15–21. All groups were provided sterile drinking water during days 1–14; from days 15–21, all groups except NC given drinking water containing 3.0% dextran sulfate sodium (DSS) to induce colitis. At the end of the experiment, all mice were anesthetized with isoflurane and sacrificed after blood collection. Colon tissues and cecal contents were collected and stored at −80 °C for further analysis.

### 2.3. Assessment of Colitis Symptoms

During DSS treatment period, the body weight, severity of diarrhea, and rectal bleeding in mice were monitored and recorded daily. The severity of colitis was evaluated using the disease activity index (DAI), calculated as the average of three parameters: body weight loss, stool consistency, and fecal blood presence [12]. The scoring criteria were as follows: Body weight loss: <1% = 0; 1–5% = 1; 5–10% = 2; 10–15% = 3; >15% = 4. Stool consistency: normal = 0; soft but still formed stool = 1; soft stool = 2; loose and unformed = 3; watery diarrhea = 4. Fecal blood: normal = 0; brown = 1; reddish = 2; visible blood traces = 3; gross rectal bleeding = 4.

### 2.4. Histopathological Analysis

The cleaned distal colon was immediately fixed in 4% paraformaldehyde for 24 h. The fixed tissues were subsequently dehydrated, paraffin-embedded, sectioned, and stained with hematoxylin and eosin (H&E). Colonic morphology was examined under a light microscope. Histopathological scoring was performed according to a previous report [12], based on the sum of three parameters: inflammatory cell infiltration, epithelial integrity, and crypt architecture.

### 2.5. Immunohistochemical Analysis of Colonic Tissue

Immunohistochemistry (IHC) was performed according to the method described by Han et al. [5]. The distribution and expression of tight junction proteins ZO-1 and Occludin, as well as the mucin MUC2, were observed under a light microscope. ImageJ software (version 1.54, NIH, Bethesda, MD, USA) was used for quantitative image analysis.

### 2.6. Biochemical Analysis

Serum levels of lipopolysaccharide (LPS) and nitric oxide (NO) were determined using ELISA kits (Shanghai Hengyuan Biotechnology Co., Ltd., Shanghai, China) and a NO assay kit (Nanjing Jiancheng Bioengineering Institute, China), respectively, following the manufacturers’ instructions. Colon tissues were homogenized in PBS, and the homogenates were centrifuged at 10,000 r/min for 10 min at 4 °C to obtain the supernatant. The concentrations of inflammatory cytokines IL-1β, IL-6, IL-10, and TNF-α were measured using ELISA kits. The activities of malondialdehyde (MDA), myeloperoxidase (MPO), and total superoxide dismutase (T-SOD) were determined with corresponding assay kits from Nanjing Jiancheng Bioengineering Institute (Nanjing, China).

### 2.7. Short-Chain Fatty Acid Analysis

The concentrations of short-chain fatty acids (SCFAs) in cecal contents were determined by gas chromatography (GC) according to the method of Zhang et al. (2024) [12], with minor modifications, with minor modifications. Briefly, 100 mg of cecal content was homogenized in 1 mL of ultrapure water and sonicated for 10 min in an ice bath. The homogenate was centrifuged at 12,000 r/min for 10 min, and 0.8 mL of the supernatant was mixed sequentially with 0.08 mL of 50% sulfuric acid and 1.6 mL of diethyl ether, followed by vortexing for 5 min. The mixture was then centrifuged at 10,000 r/min for 5 min, and the organic phase was filtered through a 0.22 μm organic membrane filter for analysis. Quantitative determination of SCFAs was performed using an Agilent 6890N (Agilent, Santa Clara, CA, USA) GC equipped with a DB-FFAP capillary column (30 m × 0.53 mm × 0.50 μm). Concentrations were calculated based on standard solutions of acetic acid, propionic acid, butyric acid, and valeric acid.

### 2.8. Gut Microbiota Analysis

Total DNA from mouse fecal samples was extracted using a Genomic DNA Kit (Omega Bio-tek, Norcross, GA, USA). DNA concentration and purity were assessed with a NanoDrop ND-2000 spectrophotometer. The bacterial 16S rRNA gene was then amplified at the V3–V4 region using primers 338F and 806R. PCR products were purified with a PCR Clean-Up Kit (LGC Bioresearch Technologies, Shanghai, China) and quantified using a Qubit 4.0 fluorometer. Sequencing was performed on the Illumina NextSeq 2000 (Illumina, San Diego, CA, SUA) platform (2 × 150 bp). Raw sequencing data were processed using the QIIME2 pipeline. The DADA2 plugin was employed for denoising, merging, and chimera filtering, and amplicon sequence variants (ASVs) were clustered for downstream analyses.

### 2.9. Statistical Analysis

All data are expressed as the mean ± standard deviation of three independent experiments. Statistical analyses were performed using SPSS 26.0 software (IBM, Armonk, NY, USA). One-way analysis of variance (ANOVA) was conducted, followed by Duncan’s multiple range test to evaluate significant differences. A significance level of *p* < 0.05 was considered statistically significant. Microbiota-related statistical analyses were conducted using R software.

## 3. Results

### 3.1. Citrus Polysaccharides Alleviate Disease Symptoms in UC Mice

**Figure 1:**
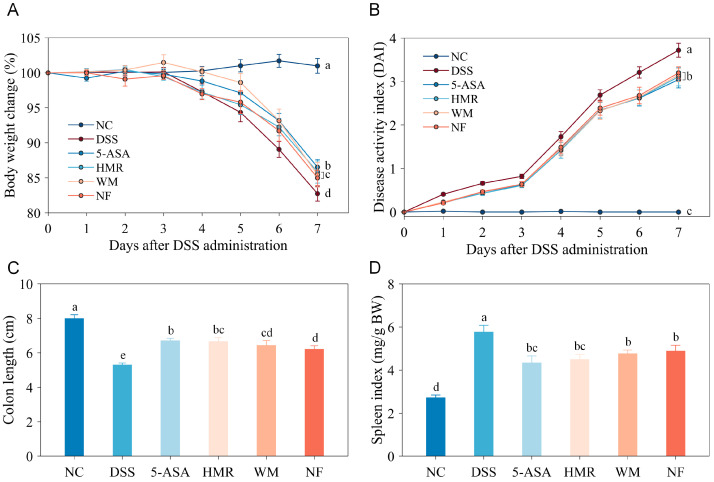
Effects of citrus polysaccharides on colitis phenotypes. (**A**) Body weight change curve; (**B**) DAI index change curve; (**C**) Colon length; (**D**) Spleen index. Groups labelled with different letters differ significantly (*p* < 0.05).

DSS-induced colitis model in mice closely mimics human ulcerative colitis (UC), sharing similar clinical features such as body weight loss, diarrhea, and rectal bleeding [13]. As shown in Figure 1A, body weight of mice decline commenced from day 4 post-modelling. By day 7, the mean body weight in the DSS group had decreased by 17.25%, while reductions of 13.47%, 14.16%, 14.55%, and 15.00% were observed in the 5-ASA, HMR, WM, and NF groups, respectively. These results indicate that citrus polysaccharide intervention effectively mitigated body weight loss in mice. To further evaluate the severity of colitis, the disease activity index (DAI) was monitored (Figure 1B). The DAI of the NC group remained at 0 throughout the experiment, whereas mice in the DSS group exhibited initial loose stools, followed by progressive rectal bleeding and body weight loss, reaching a DAI of 3.72 on day 7, confirming successful model establishment. Compared with the DSS group, all citrus polysaccharide treatments significantly reduced DAI scores (*p* < 0.05), with the greatest improvement observed in the HMR group, followed by the WM and NF groups. Colonic length serves as a crucial histological indicator reflecting the severity of inflammation [14]. Compared with the NC group (8.01 ± 0.21 cm), the DSS group exhibited significantly shortened colon length (5.31 ± 0.10 cm) with marked congestion and oedema (Figure 1C and Figure S1A). Citrus polysaccharide supplementation significantly alleviated colon shortening, particularly in the HMR group (6.66 ± 0.22 cm, *p* < 0.05). The spleen is one of the vital immune organs, responsible for the production, storage, and release of key immune cells such as macrophages and lymphocytes. Thus, the spleen index could to some extent reflect the body’s immune activity and inflammatory response levels [14]. As shown in Figure 1D, the spleen index was significantly elevated in the DSS group compared with the NC group (*p* < 0.05), indicating that DSS-induced colitis triggered splenomegaly (Figure S1B). Treatment with 5-ASA and citrus polysaccharides reduced the spleen index to varying degrees, alleviating splenic enlargement in UC mice. Collectively, citrus polysaccharide intervention effectively ameliorated UC mice symptoms, including body weight loss, increased DAI, colon shortening, and splenomegaly, with HMR showing the most pronounced protective effect.

### 3.2. Effects of Citrus Polysaccharides on Histopathology

**Figure 2:**
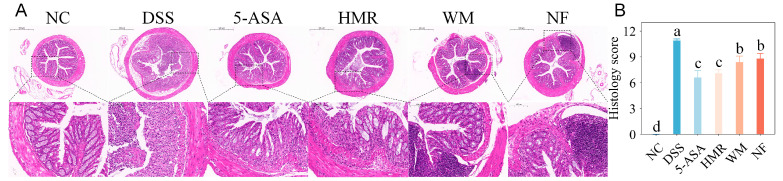
Effect of citrus polysaccharides on colonic histology. (**A**) Representative H&E images. Low-magnification images: ×40 (scale bar = 500 μm); high-magnification images: ×200 (scale bar = 100 μm). (**B**) Distal colon histological scores. Groups with different letters differ significantly (*p* < 0.05).

DSS-induced colitis is typically characterized by severe mucosal damage, crypt destruction, and infiltration of inflammatory cells, pathological features that closely resemble those observed in human ulcerative colitis [15]. As shown in Figure 2, the colonic tissue of the NC group exhibited an intact structure, with well-organized epithelial cells, clearly defined crypts architecture, and abundant goblet cells, without evidence of inflammation or necrosis. In contrast, the DSS group displayed severe injury to the colonic mucosal architecture, including epithelial cell detachment, crypt distortion or loss, and massive infiltration of inflammatory cells in the lamina propria and submucosa. The histological score was significantly elevated (10.9 ± 0.8, *p* < 0.05). Treatment with citrus polysaccharides markedly improved DSS-induced histopathological damage. In particular, the HMR group showed repaired mucosal architecture, restored crypt morphology, an increased number of goblet cells, and notably reduced inflammatory infiltration, which significantly lowered histological score (*p* < 0.05). The WM (8.4 ± 0.7) and NF (8.8 ± 0.6) groups also showed partial recovery, though focal crypt disruption and insufficient goblet cell numbers were still observed, indicating relatively weaker protective effects compared with HMR.

### 3.3. Effects of Citrus Polysaccharides on Intestinal Barrier Function

**Figure 3:**
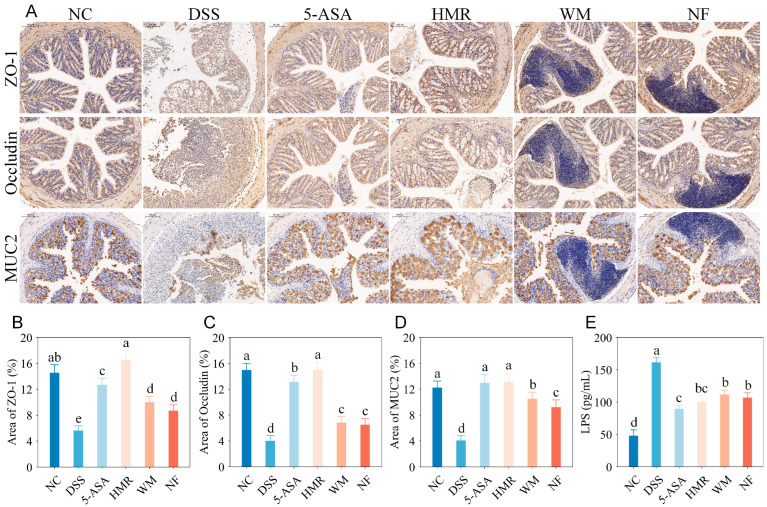
Effects of polysaccharides from citrus fruit with different mastication traits on intestinal barrier function in mice. (**A**) Representative immunohistochemistry images (scale bar = 100 μm); (**B**) ZO-1 expression area; (**C**) Occludin expression area; (**D**) MUC2 expression area; (**E**) Serum LPS levels. Groups with different letters differ significantly (*p* < 0.05).

To investigate the protective effects of citrus polysaccharides on intestinal barrier integrity, the distribution and expression of tight junction (TJ) proteins ZO-1 and Occludin, as well as mucin protein MUC2, were examined in colonic tissues by immunohistochemistry (IHC) (Figure 3). Brownish-yellow areas were considered positive expression. TJ proteins form the physical barrier of the gut, preventing luminal pathogens such as lipopolysaccharides (LPS) from penetrating the mucosal layer [5]. In the NC group, ZO-1 and Occludin were predominantly distributed along the epithelial cell membrane in a continuous arrangement. After DSS treatment, the positive expression of ZO-1 and Occludin decreased by 68.33% and 79.90%, respectively, showing discontinuous and diffuse patterns, indicating severe disruption of tight junction structure. Following citrus polysaccharide intervention, TJ proteins areas markedly recovered. HMR group exhibited the most pronounced improvement, with ZO-1 and Occludin expression increasing 2.59- and 4.02-fold, respectively. Mucin MUC2, secreted by goblet cells, constitutes the core component of the intestinal mucus layer, which forms a crucial chemical barrier against microbial and toxin invasion [5]. DSS treatment significantly reduced MUC2 expression (by 66.79%) and disrupted its continuous distribution, whereas polysaccharide treatment notably restored MUC2 distribution, with the strongest recovery observed in the HMR group. Furthermore, epithelial barrier damage often allows LPS to translocate into the bloodstream, triggering systemic inflammation [16]. Serum LPS levels in the DSS group were 2.36-fold higher than those in the NC group (*p* < 0.05), indicating compromised epithelial integrity and increased permeability. After HMR, WM, and NF, LPS levels were reduced by 37.78%, 30.87%, and 33.81%, respectively, with HMR showing the greatest reduction, further confirming its significant advantage in maintaining epithelial barrier integrity.

### 3.4. Modulation of Inflammation-Related Proteins and Cytokines by Citrus Polysaccharides

**Figure 4:**
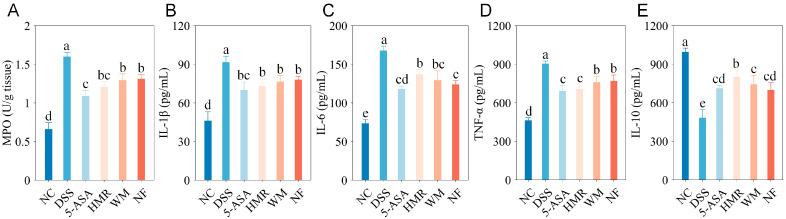
MPO and cytokine levels in mouse colon tissue. (**A**) MPO; (**B**) IL-1β; (**C**) IL-6; (**D**) TNF-α; (**E**) IL-10. Groups with different letters differ significantly (*p* < 0.05).

Myeloperoxidase (MPO) is a characteristic enzyme released by neutrophils, and its activity reflects the degree of neutrophil infiltration and activation during acute inflammation [12]. As depicted in Figure 4A, DSS treatment markedly enhanced MPO activity in colonic tissues, showing an approximately 2.42-fold increase compared with the NC group. This result is consistent with the pronounced neutrophil infiltration observed histologically (Figure 2). After citrus polysaccharide intervention, MPO activity was significantly reduced, with the strongest inhibition observed in the HMR with superior mastication trait, followed by WM, while NF showed weaker effects. This indicates that polysaccharides of citrus with different mastication traits effectively attenuate local acute inflammatory responses in the colon.

Pro-inflammatory cytokines IL-1β, IL-6 and TNF-α play a central role in the pathological process of UC, activating immune cells and disrupting the epithelial barrier, thereby exacerbating mucosal inflammatory damage [17,18]. Conversely, the anti-inflammatory cytokine IL-10 maintains immune homeostasis by suppressing pro-inflammatory signaling in macrophages [18,19]. As illustrated in Figure 4, compared with the NC group, IL-1β, IL-6, and TNF-α levels in the colonic tissue of DSS-treated mice increased by 0.98-, 1.28-, and 0.95-fold, respectively (*p* < 0.05), while IL-10 levels decreased significantly by 0.51-fold, indicating activation of the inflammatory response. Citrus polysaccharide intervention consistently suppressed pro-inflammatory cytokine to varying degrees and restored IL-10 levels. Among the treatments, HMR exhibited the most pronounced regulatory effect, characterized by significant downregulation of IL-1β and TNF-α and upregulation of IL-10. In contrast, the NF group showed lower IL-6 levels, thereby mitigating the inflammatory response to some extent.

### 3.5. Effects of Citrus Polysaccharides on Oxidative Stress in UC Mice

**Figure 5:**
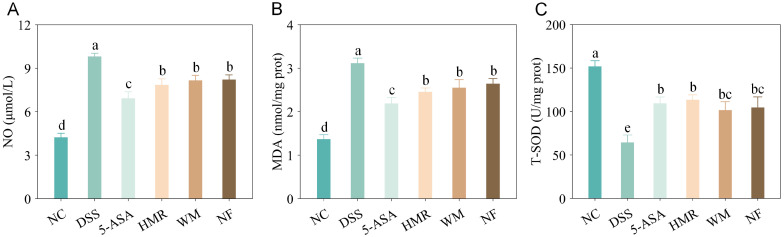
Effects of citrus polysaccharides on oxidative stress. (**A**) NO content in mice serum; (**B**) MDA content in colon; (**C**) T-SOD activity in colon. Groups with different letters differ significantly (*p* < 0.05).

Oxidative stress is closely associated with inflammation, as activated immune cells during the inflammatory response produce excessive reactive oxygen species (ROS) and nitric oxide (NO), leading to lipid peroxidation, cellular damage, and disruption of epithelial barrier function [16]. To investigate the regulatory effects of citrus polysaccharides on oxidative stress in UC mice, serum NO levels, colonic MDA content, and T-SOD activity were measured (Figure 5). Compared with the NC group, DSS treatment significantly increased serum NO levels. Following intervention, NO concentrations were significantly reduced, and the 5-ASA group exhibited a significantly lower NO level than the citrus polysaccharide groups (*p* < 0.05), indicating a stronger antioxidative capacity of 5-ASA. Among the citrus polysaccharide treatments, the HMR group demonstrated the greatest reduction in NO, suggesting a superior antioxidant potential compared with WM and NF.

MDA, a major product of lipid peroxidation, reflects the extent of ROS-mediated tissue injury [17]. DSS administration markedly elevated MDA levels in colonic tissues, whereas treatment with HMR, WM, and NF reduced MDA concentrations by 29.77%, 21.27%, and 15.21%, respectively, indicating that citrus polysaccharides mitigated oxidative damage in the colon. In addition, T-SOD activity reflects the organism’s ability to eliminate superoxide radicals [17]. Compared with the DSS group, both 5-ASA and citrus polysaccharide treatments significantly enhanced T-SOD activity, with the HMR group showing the highest increase (0.76-fold, *p* < 0.05), demonstrating its advantage in maintaining redox homeostasis.

### 3.6. Effects of Citrus Polysaccharides on SCFAs

**Figure 6:**
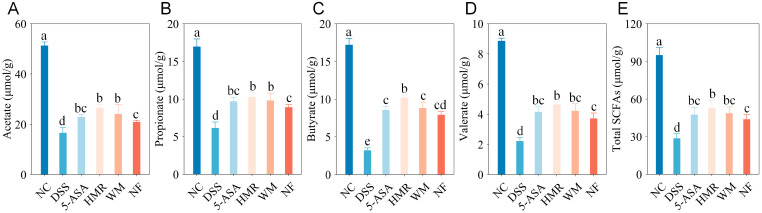
SCFA levels in mice. (**A**) Acetate; (**B**) Propionate; (**C**) Butyrate; (**D**) Valerate; (**E**) Total SCFAs. Groups with different letters differ significantly (*p* < 0.05).

Short-chain fatty acids (SCFAs) are the primary metabolites produced by gut microbiota fermentation of dietary polysaccharides. They can alleviate colitis by inhibiting the synthesis of inflammation-related cytokines and enhancing the expression of TJ proteins and mucins [20]. As shown in Figure 6, compared with the NC group, the DSS group exhibited substantial reductions in acetate, propionate, butyrate, valerate, and total SCFA levels by 67.57%, 63.65%, 81.28%, 75.00%, and 69.86%, respectively, indicating markedly impaired gut microbial metabolic function. Following treatment with 5-ASA and citrus polysaccharides, SCFA levels were significantly restored. HMR showed the most pronounced improvement, with acetate, propionate, butyrate, valerate, and total SCFA levels increased by 0.60-, 0.67-, 2.17-, 1.09-, and 0.80-fold, respectively, compared with the DSS group. The WM group exhibited the second highest increase, while the NF group displayed a relatively weaker effect.

### 3.7. Effects of Citrus Polysaccharides on Gut Microbiota

**Figure 7:**
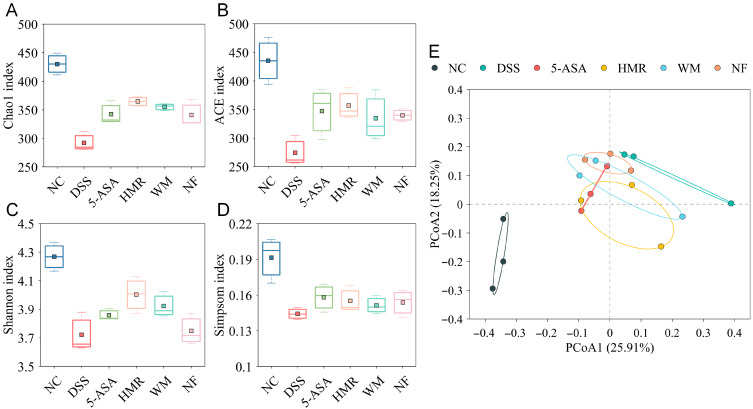
Effects of citrus polysaccharides on gut microbiota diversity. (**A**) Chao1; (**B**) ACE; (**C**) Shannon; (**D**) Simpsom; (**E**) PCoA plot based on Bray–Curtis distances.

Based on findings that citrus polysaccharides promote SCFAs production, the regulatory effects of polysaccharides from citrus fruit with different mastication traits on mice gut microbiota were investigated. As illustrated in Figure 7, DSS induction significantly decreased α-diversity indices (Chao1, ACE, Shannon, and Simpson), indicating that colitis disrupted gut microbial homeostasis. After citrus polysaccharide intervention, microbial diversity was restored to varying degrees, with the most notable improvement observed in the HMR group, which exhibited enhanced microbial richness and evenness. β-diversity analysis confirmed this trend. PCoA plot (Figure 7E) revealed clear separation between the DSS and NC groups, suggesting marked alterations in community structure. In contrast, the 5-ASA and citrus polysaccharide groups shifted closer to the NC group, with the HMR group clustering most closely with NC, suggesting that citrus polysaccharides could ameliorate microbial dysbiosis in UC mice.

**Figure 8:**
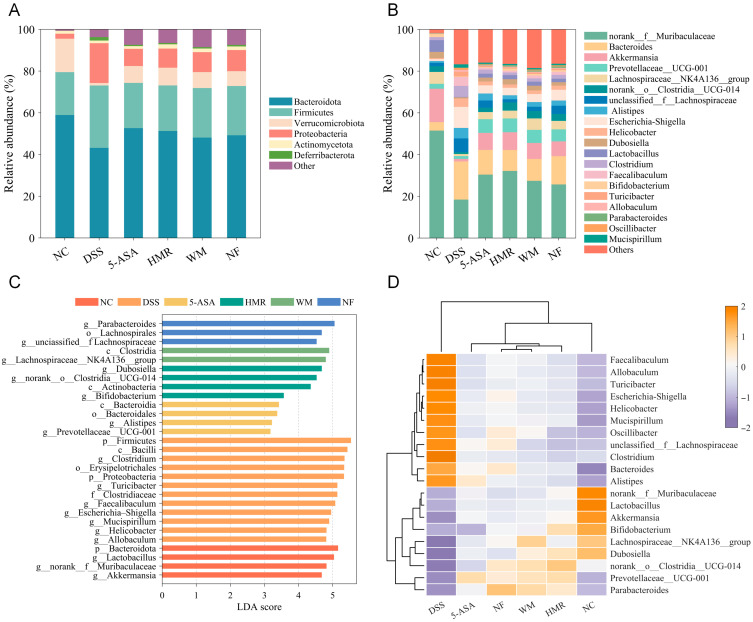
Effects of citrus polysaccharides on gut microbiota composition. (**A**) Gut microbiota structure at phylum level; (**B**) Gut microbiota structure at genus level; (**C**) LEfSe analysis of differentially abundant taxa (LDA > 3); (**D**) Heatmap of microbial communities at genus level.

At the phylum level (Figure 8A), Proteobacteria and Firmicutes were significantly enriched in DSS-induced UC mice, whereas the relative abundance of Bacteroidota decreased. Citrus polysaccharide treatment reduced relative abundance of Proteobacteria and increased the proportions of Bacteroidota and Verrucomicrobiota, suggesting their potential to remodel the gut microbial community. At the genus level (Figure 8B), the top 20 most abundant taxa showed marked compositional shifts. Compared with the NC group, the DSS group exhibited significantly increased relative abundances of *Bacteroides*, *unclassified_f Lachnospiraceae*, *Alistipes*, *Escherichia–Shigella*, *Helicobacter*, *Clostridium*, and *Faecalibaculum*, while beneficial genera such as *norank_f_Muribaculaceae*, *Akkermansia*, *Prevotellaceae_UCG-001*, *Lachnospiraceae_NK4A136_group*, *Dubosiella*, and *Lactobacillus* were markedly reduced. Citrus polysaccharide intervention reversed this imbalance to varying degrees, decreasing the relative abundance of potentially pro-inflammatory bacteria and promoting the recovery of beneficial taxa associated with SCFAs production and mucosal homeostasis. HMR with superior mastication trait markedly enhanced the abundance of *norank_f_Muribaculaceae* and *Akkermansia*, whereas NF group retained higher proportions of *Bacteroides* and *unclassified_f_Lachnospiraceae*. To reveal the differences in characteristic taxa among groups, LEfSe analysis with an LDA score > 3 was performed (Figure 8C). Results revealed that the dominant genera in HMR were *Dubosiella*, *Bifidobacterium*, and *norank_o_Clostridia_UCG-014*; it was *Lachnospiraceae_NK4A136_group* in WM; and in NF, it was *Parabacteroides*. The heatmap of microbial composition (Figure 8D) further demonstrated that the microbial profile of the HMR group was most similar to that of the NC group, indicating greater potential for restoring intestinal homeostasis.

### 3.8. Correlation Analysis Among Gut Microbiota, SCFAs, and Colitis-Related Indicators

**Figure 9:**
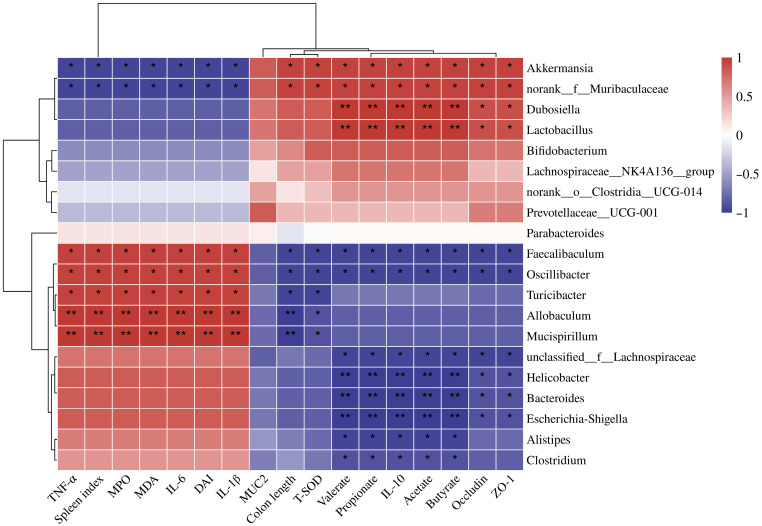
Spearman correlation analysis between gut microbiota, SCFAs, and indicators associated with DSS-induced colitis. Red and blue represent positive and negative relationships, respectively, and the gradient of colour corresponds to the magnitude of the correlation. Levels of significance are denoted by * (*p* < 0.05) and ** (*p* < 0.01).

To identify key microbial communities potentially associated with disease severity, correlation analyses were performed between gut microbiota composition and colitis-related indicators (Figure 9). The results revealed that the abundances of *Faecalibaculum*, *Oscillibacter*, and *Escherichia–Shigella* were positively correlated with inflammatory and oxidative stress indicators, including TNF-α, IL-6, IL-1β, MPO, MDA, DAI, and spleen index, while showing significant negative correlations with colon length, T-SOD activity, SCFA levels, IL-10, Occludin, and ZO-1 expression. Similarly, *Helicobacter* and *unclassified_f_Lachnospiraceae* exhibited comparable correlation patterns, suggesting that these bacteria may exacerbate inflammatory responses and mucosal injury in UC mice. In contrast, beneficial bacteria such as *Akkermansia*, *norank_f_Muribaculaceae*, *Dubosiella*, and *Lactobacillus* were negatively correlated with pro-inflammatory cytokines and oxidative stress markers, but positively correlated with colon length, IL-10, acetate, propionate, butyrate, valerate, and TJ proteins. These findings suggest that citrus polysaccharides may alleviate adverse symptoms in UC mice by promoting the proliferation of the aforementioned beneficial bacteria.

## 4. Discussion

DSS-induced mice exhibited typical pathological features of UC, including body weight loss, colon shortening, increased DAI and spleen index, alongside colonic tissue displaying marked mucosal damage, crypt disruption, and inflammatory cell infiltration. Citrus polysaccharide interventions significantly alleviated UC symptoms, among which HMR, characterized by superior mastication trait, showed the most pronounced improvement, followed by WM, while NF exhibited the weakest effect. This difference may relate to their composition and structure. HMR contains a higher proportion of pectin, whereas NF has higher cellulose content and crystallinity but a lower level of pectin (Figure S2), which might limit its bioavailability. Previous studies have recommended high-fiber diets for patients with colitis with rectal injury [21], while UC patients are advised to consume diets rich in soluble dietary fiber [22]. This aligns with our finding that HMR, which contains more pectin, showed the most pronounced ameliorative effect on UC. At the histological level, citrus polysaccharide treatment significantly restored the colonic crypt structure and goblet cell abundance. Goblet cells maintain the intestinal mucus barrier by secreting mucin MUC2, while TJ proteins determine intestinal epithelial integrity and permeability. Abnormal expression of TJ proteins and disruption of the mucus layer in UC mice aggravate disease progression [5]. Supplementation with citrus polysaccharides restored the expression of ZO-1 and Occludin, as well as MUC2, indicating their protective role in maintaining intestinal barrier function. The HMR group exhibited the most pronounced recovery effects, potentially attributable to the pectin-rich HMR being more easily fermented by gut microbiota. This promotes the production of SCFAs such as butyrate, thereby stimulating goblet cell differentiation and enhancing energy metabolism, ultimately repairing the mucus layer [23]. In addition, galacturonic acid within pectin may indirectly maintain ZO-1 and Occludin integrity by suppressing inflammatory responses and slowing TJ proteins degradation [24]. The reduction in serum LPS levels further confirmed the restoration of intestinal barrier function in UC mice.

Inflammatory cytokines play essential roles in regulating intestinal barrier and epithelial function. Increased intestinal permeability leads to dysregulation of the immune response to intestinal bacterial antigens, thereby triggering intestinal inflammation [4]. Citrus polysaccharides downregulated the levels of pro-inflammatory cytokines IL-1β, IL-6, and TNF-α while upregulating the expression of the anti-inflammatory cytokine IL-10 in UC mice. Among them, HMR exhibited the most pronounced anti-inflammatory effect, whereas NF showed relatively weaker activity. This phenomenon is closely associated with the structure of polysaccharides. HMR contains a higher overall pectin content, with a greater proportion of homogalacturonan (HG) regions, and relatively long, weakly aggregated RG-I chains (Table S2) [11], rendering it more susceptible to degradation by gut microbiota. This degradation yields galacturonic acid (GalA) fragments generating immunomodulatory activity. GalA oligosaccharides derived from HG backbones have been demonstrated to bind the TLR4 receptor, thereby inhibiting LPS-mediated IL-1β and TNF-α signaling [25,26]. Furthermore, galactose residues located in the RG-I side chains can interact with the Galectin-3 receptor, promoting macrophage M2 polarization and enhancing IL-10 secretion [27]. These explain the significant advantage of HMR in regulating the IL-1β, TNF-α, and IL-10 cytokine balance. In contrast, NF contains higher levels of cellulose with greater crystallinity (Figure S3), forming a compact matrix that limits the exposure and biodegradability of HG backbones, thereby weakening its regulatory potential on TLR4-associated pathways. Nevertheless, NF pectin is characterized by a markedly higher arabinose content (Table S1), giving rise to highly branched RG-I side chains with pronounced molecular aggregation [11]. Previous studies have reported that RG-I structures enriched in arabinose side chains may selectively suppress IL-6 secretion in macrophages [28], which is consistent with the markedly lower IL-6 levels observed in the NF-treated mice in this study. Similarly, Wu et al. [16] demonstrated that lemon pectin, rich in arabinose side chains, significantly suppressed IL-6 expression. Therefore, although NF contains relatively low pectin levels, its abundant arabinose side chain structure confers immune signalling regulatory activity, contributing to a partial alleviation of inflammation in UC mice. Tissue inflammation is often accompanied by excessive production of reactive oxygen species, which aggravates cellular oxidative damage [4,16]. The results showed that citrus polysaccharides significantly reduced serum NO and colonic MDA levels while increasing T-SOD activity, indicating a protective effect against oxidative injury in colonic tissues. Among these, HMR demonstrated optimal efficacy, potentially attributable to its high galacturonic acid content and consequent free radical scavenging capacity [29].

Citrus polysaccharides are resistant to digestion in the stomach and small intestine and are primarily degraded and metabolized by colonic microbiota to produce metabolites such as SCFAs [30]. In DSS-induced UC mice, the levels of SCFAs were markedly reduced, which is consistent with previous findings [4,12]. Citrus polysaccharide intervention restored these levels, with the HMR group exhibiting the most pronounced increases in butyrate and propionate, consistent with trends observed in our previous in vitro colonic fermentation results. Butyrate has been reported to stimulate IL-10 expression and promote epithelial repair, whereas propionate contributes to the regulation of T-cell homeostasis and immune balance, thereby alleviating inflammatory responses [14]. Furthermore, the higher valerate content in HMR may suppress inflammatory responses and regulate immune homeostasis by upregulating IL-10 and inhibiting B-cell apoptosis [31]. In contrast, NF, characterized by a stable structure and high crystallinity, was less accessible to microbial degradation, resulting in limited SCFAs production, which was consistent with its weaker anti-inflammatory effect.

The gut microbiota plays a pivotal regulatory role in the onset and remission of colitis. DSS induction caused intestinal microecological imbalance in UC mice, characterized by a significant decrease in α-diversity and reconstruction of the microbial community structure, indicating disruption of intestinal homeostasis. Compared with the NC group, the relative abundances of *Bacteroides*, *Alistipes*, *Escherichia–Shigella*, and *Helicobacter* were significantly increased in the DSS group. These genera are closely associated with intestinal barrier disruption, infectious diarrhea, inflammation, and gastrointestinal injury [32,33,34]. Citrus polysaccharide intervention effectively suppressed the proliferation of potential pro-inflammatory bacteria such as *Escherichia–Shigella* and *Helicobacter*, while promoting the growth of beneficial bacteria including *Akkermansia*, *norank_f_Muribaculaceae*, *Lachnospiraceae_NK4A136_group*, and *Prevotellaceae_UCG-001*. These changes were closely associated with intestinal barrier repair, SCFAs production, and immune homeostasis. *Akkermansia* exerts anti-inflammatory effects by promoting mucus renewal and inducing IL-10 expression through regulatory T cell differentiation [7]; *Muribaculaceae* produces acetate and propionate to enhance host immune function [18]; *Lachnospiraceae* enhances epithelial barrier integrity and suppresses inflammation by promoting SCFAs production [35]. The pectin-rich HMR significantly enriched beneficial bacteria such as *norank_f_Muribaculaceae* and *Akkermansia*, whereas the NF group exhibited higher relative abundances of *Bacteroides* and *unclassified_f_Lachnospiraceae*. This disparity likely stems from the higher cellulose content, stronger crystallinity, and lower pectin proportion of NF, which restrict its microbial degradability [30]. Consistent with the in vivo findings in mice, our previous in vitro colonic fermentation experiment also confirmed that HMR exhibited higher fermentability. LEfSe analysis further revealed that polysaccharides from citrus fruit with different mastication traits exhibited distinct patterns of gut microbiota modulation. The pectin-rich HMR selectively promoted the proliferation of *Dubosiella* and *norank_o_Clostridia_UCG-014*, whereas WM enriched *Lachnospiraceae_NK4A136_group* and NF favored the growth of *Parabacteroides*. Similarly, pectins from mature and unripe kiwifruits selectively promoted the growth of specific bacterial taxa due to structural differences, thereby exhibiting distinct effects in alleviating colitis [7]. Spearman correlation analysis demonstrated that *Akkermansia* and *norank_f_Muribaculaceae* exhibited significant positive correlations with SCFAs, IL-10, and tight junction proteins, whilst showing negative correlations with IL-1β, TNF-α, MDA, and DAI. These findings suggest that citrus polysaccharides may ameliorate colitis by reshaping the gut microbial community, thereby enhancing SCFA production, suppressing inflammation, alleviating oxidative stress, and repairing mucosal barrier integrity.

## 5. Conclusions

This study systematically evaluated the ameliorative effects of polysaccharides (HMR, WM, and NF) extracted from citrus fruits with different mastication traits on DSS-induced ulcerative colitis. The results demonstrate that citrus polysaccharides alleviate colitis symptoms. Among them, HMR with superior mastication traits exerts the strongest protective effect by significantly restoring the expression of IL-10, ZO-1, Occludin, and MUC2, while suppressing excessive TNF-α production, thereby promoting mucosal barrier repair and mitigating inflammatory responses. In contrast, NF, which contains abundant arabinose side chains, showed a greater ability to downregulate IL-6 expression. Moreover, citrus polysaccharides promote the enrichment of beneficial bacteria such as *Akkermansia* and *norank_f_Muribaculaceae*, enhance SCFA levels (particularly propionate and butyrate), and suppress the proliferation of pathogenic bacteria including *Escherichia*–*Shigella* and *Helicobacter*. LEfSe analysis further revealed that polysaccharides from citrus cultivars with different mastication traits selectively modulate gut microbiota composition, with pectin-rich HMR showing a stronger capacity to restore microbial homeostasis. Although the precise mechanisms underlying the structural dependence of these polysaccharides on UC mitigation warrant further investigation, this study provides a scientific basis for the dietary interventions for inflammatory bowel disease using citrus with different mastication traits, and provides relevant insight for dietary interventions and citrus breeding strategies.

## Data Availability

The original contributions presented in this study are included in the article/supplementary material. Further inquiries can be directed to the corresponding author.

## Supplementary Materials

The following supporting information can be downloaded at: https://www.mdpi.com/article/10.3390/foods15010052/s1, Figure S1: Representative images of the colon and spleen; Figure S2: Relative percentages of polysaccharide fractions in citrus with different mastication traits; Table S1: Monosaccharide composition of pectin in citrus fruit; Table S2: Percentages of HG and RG-I, and DBr for pectin fractions from citrus fruit; Figure S3: (A) XRD patterns of cellulose; (B) Crystallinity index and thickness of crystallites of cellulose.
