# Supplementary material for: Polysaccharides from Citrus Fruit with Different Mastication Traits Ameliorate DSS-Induced Ulcerative Colitis by Restoring Intestinal Barrier Function and Microbiota Balance
Source: Foods. 2025 Dec 24;15(1):52. doi: 10.3390/foods15010052 (PMC12785777; doi:10.3390/foods15010052)
Supplement: Supplementary file 1 [file foods-15-00052-s001.zip › foods-4025080-supplementary.pdf]

Supplementary Information

Polysaccharides from Citrus Fruit with Different Mastication Traits Ameliorate DSS-Induced Ulcerative Colitis by Restoring Intestinal Barrier Function and Microbiota Balance

Jieqiong Yao 1,2,3 and Siyi Pan 1,2,3,*

1 College of Food Science and Technology, Huazhong Agricultural University, Wuhan 430070, China; yaojieqiong2021@webmail.hzau.edu.cn

2 Key Laboratory of Environment Correlative Dietology, Ministry of Education, Huazhong Agricultural University, Wuhan 430070, China

3 Hubei Key Laboratory of Fruit & Vegetable Processing & Quality Control, Huazhong Agricultural University, Wuhan 430070, China

* Correspondence: pansiyi@mail.hzau.edu.cn; Tel.: +86-135-5402-9828

SI. Representative images of the colon and spleen

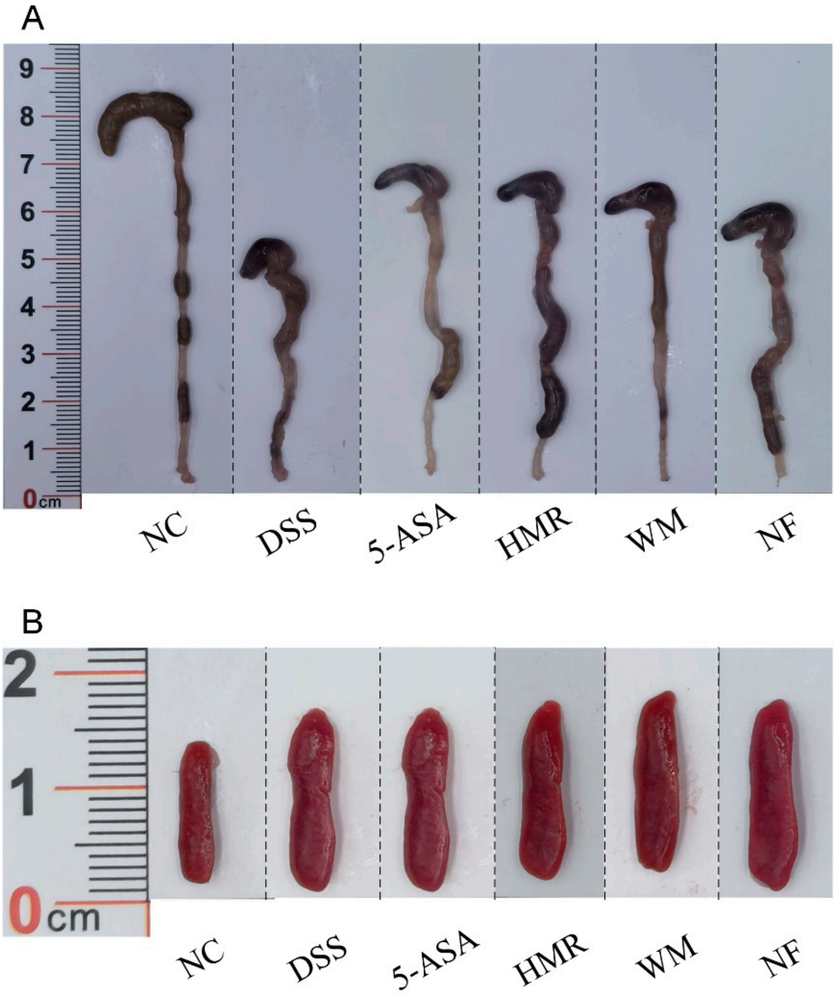

Figure S1. (A) Representative colon image; (B) representative spleen image.

SII. Content of fractions of the polysaccharide of citrus fruit pulp

Figure S2 shows the relative content of polysaccharide fractions in HMR, WM and NF [1]. The WSF percentage was highest in HMR (46.9%), followed by WM (46.1%) and only 36.2% in NF. In addition, CEL content was significantly higher in NF (24.8%) compared to HMR (16.0%) and WM (17.3%). This higher CEL level in NF is likely contributing to the denser aggregation of parenchyma tissue cellulose microfibrils and enhancing its mechanical power.

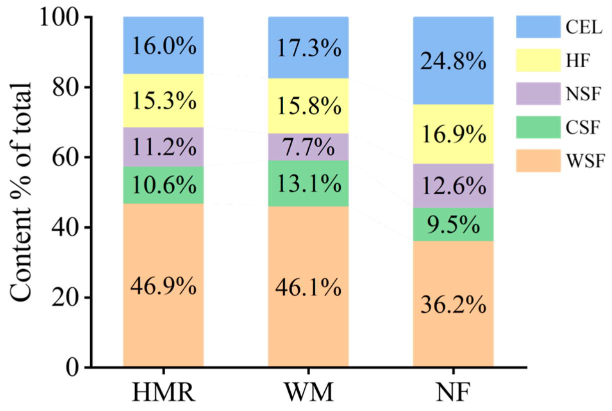

Figure S2. Relative percentages of polysaccharide fractions in citrus with different mastication traits [1]. WSF, water-soluble pectin fraction; CSF, chelator-soluble pectin fraction; NSF, Na_2CO_3-soluble pectin fraction; HF, hemicellulose fraction; CEL, cellulose.

SIII. Monosaccharide composition

Table S1. Monosaccharide composition of pectin in citrus fruit [1].

Sample		Content in sample (mol%)								
		Man	Rha	GlcA	GalA	Glc	Gal	Xyl	Ara	Fuc
	HMR	2.53 ± 0.57	9.51 ± 0.61^{a}	0.83 ± 0.34^{a}	27.43 ± 2.91^{a}	1.16 ± 0.30^{a}	13.89 ± 0.99^{a}	0.52 ± 0.25	28.76 ± 4.12^{a}	0.18 ± 0.02^{a}
WSF	WM	nd	7.21 ± 0.39^{a}	0.28 ± 0.08^{b}	33.26 ± 1.81^{a}	1.03 ± 0.17^{a}	16.86 ± 0.71^{a}	nd	38.15 ± 2.08^{a}	0.12 ± 0.05^{a}
	NF	nd	6.68 ± 0.41^{a}	0.35 ± 0.05^{b}	39.48 ± 3.99^{a}	0.67 ± 0.16^{a}	15.74 ± 0.94^{a}	nd	37.00 ± 3.10^{a}	0.08 ± 0.02^{b}
	HMR	nd	15.68 ± 0.01^{a}	0.44 ± 0.04	28.02 ± 1.74^{a}	1.14 ± 0.02^{a}	14.29 ± 0.38^{c}	1.93 ± 0.10^{a}	38.49 ± 1.48^{c}	nd
CSF	WM	nd	11.44 ± 0.35^{b}	nd	18.69 ± 2.17^{b}	5.19 ± 5.36^{a}	16.97 ± 0.64^{b}	1.78 ± 0.21^{a}	45.93 ± 2.21^{b}	nd
	NF	nd	8.88 ± 0.18^{c}	nd	16.14 ± 0.82^{b}	1.07 ± 0.75^{a}	19.08 ± 0.17^{a}	nd	54.82 ± 0.41^{a}	nd
	HMR	nd	11.58 ± 0.84^{a}	1.60 ± 0.19^{a}	19.30 ± 1.06^{a}	6.26 ± 6.61^{a}	21.05 ± 1.42^{b}	1.23 ± 0.34^{a}	38.84 ± 2.99^{c}	0.15 ± 0.20
NSF	WM	nd	9.50 ± 0.28^{b}	0.39 ± 0.04^{b}	11.73 ± 0.52^{b}	0.62 ± 0.04^{a}	22.97 ± 0.16^{b}	0.72 ± 0.16^{b}	54.06 ± 0.30^{b}	nd
	NF	nd	7.26 ± 0.63^{c}	0.39 ± 0.02^{b}	7.74 ± 0.39^{c}	0.24 ± 0.02^{a}	25.50 ± 0.78^{a}	nd	58.89 ± 1.14^{a}	nd

Note: Different letters within a column denote significant differences. Man, mannose; Rha, rhamnose; GlcA, glucuronic acid; GalA, galacturonie acid; Glc, glucose; Gal, galactose; Xyl, xylose; Ara, arabinose; Fuc, fucose.

SIV. Structure of pectin fractions

Table S2. Percentages of HG, RG-I, and DBr for pectin fractions from citrus fruit [1].

Sample		HG (%)	RG-I (%)	DBr
	HMR	22.55 ± 2.64^{a}	76.60 ± 2.68^{a}	4.50 ± 0.31^{b}
WSF	WM	27.26 ± 2.19^{a}	72.61 ± 2.16^{a}	7.63 ± 0.05^{a}
	NF	33.14 ± 1.44^{a}	66.78 ± 2.44^{a}	8.00 ± 0.77^{a}
	HMR	12.54 ± 1.36^{a}	85.50 ± 1.86^{b}	3.37 ± 0.12^{c}
CSF	WM	7.58 ± 0.80^{b}	90.54 ± 1.60^{a}	5.50 ± 0.14^{b}
	NF	2.25 ± 0.55^{b}	92.66 ± 0.95^{a}	8.32 ± 0.16^{a}
	HMR	8.39 ± 0.10^{a}	90.13 ± 0.47^{c}	5.18 ± 0.24^{c}
NSF	WM	2.25 ± 0.20^{b}	97.02 ± 0.92^{b}	8.11 ± 0.21^{b}
	NF	0.48 ± 0.05^{c}	99.5 ± 0.95^{a}	11.69 ± 0.19^{a}

SV. X-ray diffraction analysis

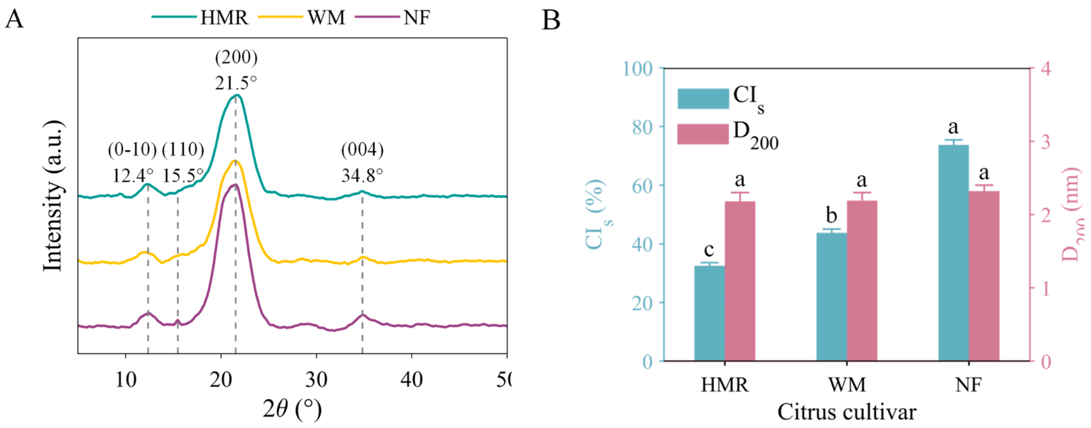

Figure S3. (A) XRD patterns of cellulose; (B) crystallinity index and thickness of crystallites of cellulose [1].

References

1. Yao, J.; Yang, C.; Shi, K.; Liu, Y.; Xu, G.; Pan, S. Effect of Pulp Cell Wall Polysaccharides on Citrus Fruit with Different Mastication Traits. *Food Chemistry* **2023**, *429*, 136740, doi:10.1016/j.foodchem.2023.136740.
